# Spatial equity-oriented accessibility assessment and optimal allocation of older adult care facilities in Beijing’s central urban area

**DOI:** 10.3389/fpubh.2026.1779461

**Published:** 2026-04-14

**Authors:** Jianyi Huang, Hongjie Wang

**Affiliations:** College of Applied Arts and Science, Beijing Union University, Beijing, China

**Keywords:** older adults, spatial accessibility, care facilities, optimal allocation, Beijing

## Abstract

**Introduction:**

Population aging is a pressing global public health challenge. In aging-concentrated urban central areas, accelerated population aging has worsened the mismatch between supply and demand, irrational spatial layout, and inadequate accessibility of older adult care resources.

**Methods:**

To improve public service equity, this study took Beijing’s central urban area as the research object. Using data from the 2018 Beijing Special Census on Targeted Assistance for older adults and the 2020 Full-Coverage Census of older adult care Facilities, facility accessibility was evaluated using an improved Ga2SFCA method. The number of required facilities was determined via K-means clustering combined with the elbow rule, and optimal locations was verified using particle swarm optimization and remote sensing images.

**Results:**

The results showed that: (1) Accessibility of older adult care facilities was spatially imbalanced, with some areas lacking coverage or only having single-type facilities. (2) Community service stations were the most accessible, especially in Dongcheng and Xicheng districts; older adult care centers had the lowest accessibility; while older adult care institutions performed better in peripheral zones.(3) We proposed an optimal allocation plan with 16 new facilities in underserved residential areas with high concentrations of older adults.

**Discussion:**

This integrated framework improves the precision of accessibility evaluation and site optimization. The findings offer empirical support for equitable older adult care policy-making in Beijing, helping enhance service equity and sustainable development.

## 1 Introduction

Population aging has become a global public health challenge, with profound implications for the equity and efficiency of public service provision. In China, Beijing, as a national political and cultural center, is experiencing an accelerated aging process, which poses a critical constraint to its goal of building a world-class harmonious and livable metropolis. A typical feature of China’s aging trajectory is “aging before affluence” (1), where the rapid growth of the older adults outpaces economic development, leading to significant regional disparities in the demand for and allocation of older adult care resources. With the spatial–temporal differentiation of population aging, core issues such as mismatches between older adult care resources and the older adults, underutilization of facilities, and irrational spatial layout have become increasingly prominent (2). Older adult care facilities, including community older adult care service stations, older adult care institutions, and older adult care centers, are key carriers of public older adult care services (3). The spatial equity and accessibility of these facilities directly affect the health and well-being of the older adults, making them a core concern in public health and urban planning. Therefore, accurately evaluating the spatial accessibility of older adult care facilities and optimizing their siting to achieve balanced resource allocation are essential for addressing the aging challenge and advancing public service equity in Beijing.

Spatial accessibility is a fundamental indicator for assessing the rationality of public service facility distribution (4), as it quantifies the ease with which individuals can access services while accounting for spatial barriers (5). Since the concept was first proposed in 1959, extensive research has been conducted globally, with common methods including potential modeling, the two-step floating catchment area (2SFCA) method, the cumulative opportunity approach, and the shortest distance method (6, 7). Among these, the 2SFCA method is widely adopted due to its ability to integrate both supply and demand factors and their spatial interactions, yielding more reliable results than alternative approaches (8). Accessibility analysis has been applied across diverse public service fields, such as healthcare, education, and older adult care, with studies covering various geographic scales and contexts (9–11). For example, Ngui and Apparicio (12) demonstrated the superiority of improved 2SFCA in evaluating healthcare accessibility for vulnerable groups, while Kocatepe et al. (13) applied accessibility models to optimize emergency shelter siting for older adults, highlighting its value in public health-oriented spatial planning. In China, research on public facility accessibility emerged in the early 21st century, focusing primarily on model improvement and parameter optimization to enhance applicability (14). In the field of older adult care, scholars have increasingly applied accessibility models to assess facility distribution. Cheng and Huang (15) used the Gaussian two-step floating catchment area (Ga2SFCA) method to analyze the correlation between older adult care facility accessibility and older adults distribution in central Shanghai. Wu et al. (16) optimized the traditional potential model with a demand threshold to evaluate institutional older adult care facilities in Shanghai’s Fengxian District. Tao et al. (17) also explored older adult care facility accessibility in Beijing using an improved 2SFCA method.

Despite these advancements, existing studies still have gaps relevant to public health practice: First, regarding assessment accuracy, most studies use administrative divisions as the basic unit for demand measurement, ignoring internal spatial differences in older adult care needs and leading to inaccurate evaluations (18–20); Conventional 2SFCA adopts a stepwise distance decay function, which fails to continuously and accurately reflect the attenuation of service capacity with distance (21). Although the Ga2SFCA method addresses this limitation by employing a Gaussian function to characterize distance decay (3, 22), thus avoiding the use of alternative distance decay functions with attenuation rates that change monotonically with increasing distance, it has rarely been integrated with fine-grained demand measurement approaches in existing older adult care facility research; Consequently, single-method approaches often struggle to capture the realistic, micro-scale accessibility landscape required for effective urban interventions. Second, regarding siting optimization, prior literature often treats facility quantity and location as separate problems or relies on single-objective algorithms. These single-method approaches lack a systematic mechanism to simultaneously determine the optimal number of facilities based on demand clustering and pinpoint their precise locations. Consequently, the resulting schemes may achieve theoretical coverage but fail to maximize spatial equity in practice. Moreover, few studies have targeted Beijing’s central urban area—a region with high population density, complex spatial structure, and acute older adult care demand—further limiting the practical guidance of research results for local public health and urban planning policies.

This study proposed an integrated research framework combining grid GIS, Ga2SFCA, K-means clustering, and particle swarm optimization (PSO) algorithm responding to the public health demand for equitable older adult care services. The specific technical route is as follows: (1) Taking Beijing’s central urban area as the study area, grid GIS was used to replace traditional administrative divisions as the basic unit for demand measurement; (2) The Ga2SFCA method was adopted to calculate the accessibility of three types of older adult care facilities (older adult care institutions, older adult care centers, and community older adult care service stations), with Tyson polygon used to determine the service radius of different facilities; (3) Raster points with low accessibility and non-zero older adults were identified as optimization areas. The K-means clustering algorithm combined with the elbow method was used to determine the number of new older adult care facilities, and the PSO algorithm is applied to optimize their siting; (4) Feasibility analysis of the proposed sites was conducted using remote sensing images to enhance the practical applicability of the optimization scheme. This study aims to assess the spatial accessibility of older adult care facilities in Beijing’s central urban area, develop practically viable siting optimization strategies, and provide empirical data and decision support for local equitable older adult care policies, thereby facilitating sustainable service development, enhancing older adults’ life quality, and advancing service equity in alignment with public health and urban sustainability goals.

## 2 Materials and methods

### 2.1 Study area

**Figure 1:**
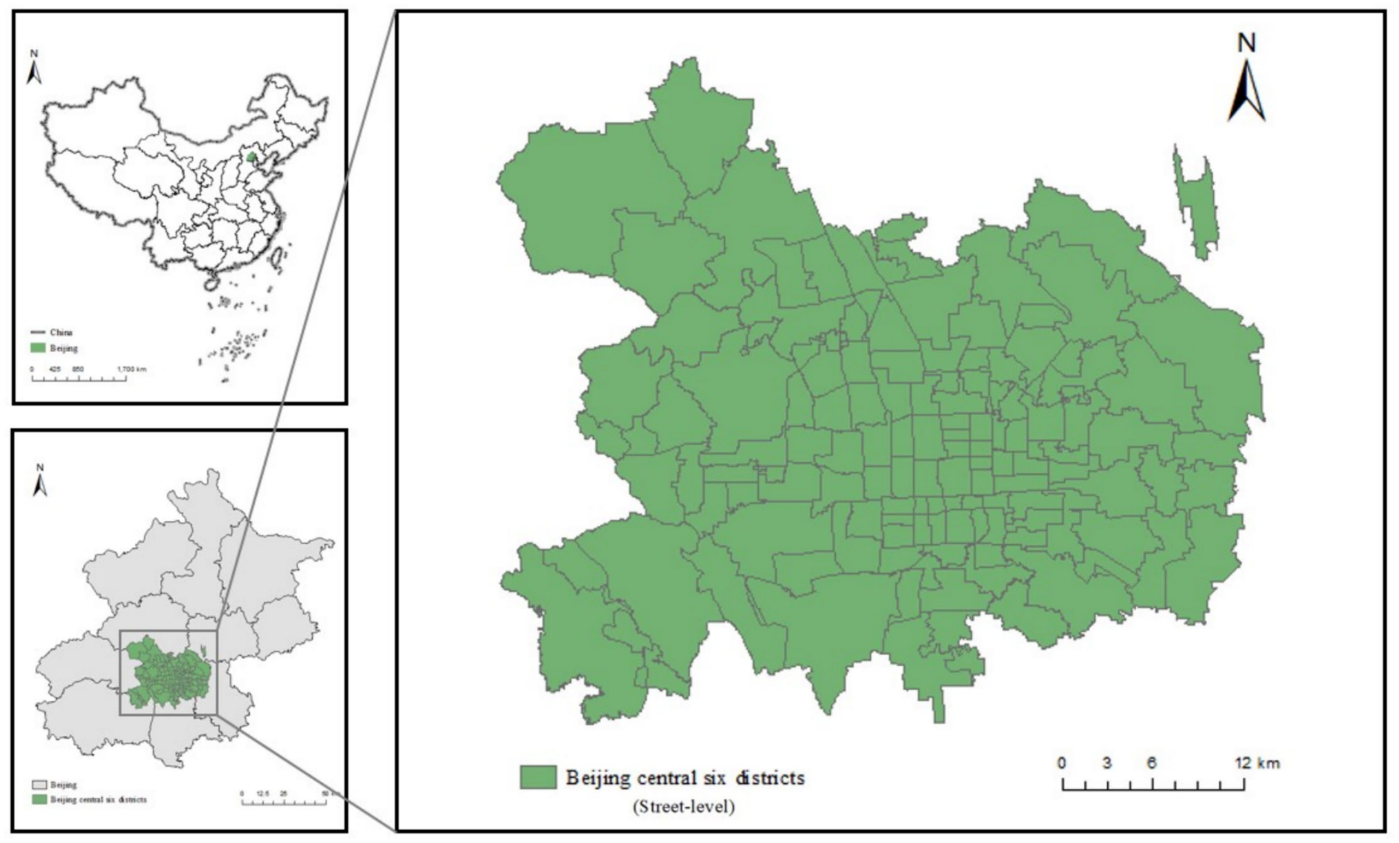
Study area. The map of China is drawn based on the standard map from the State Bureau of Surveying and Mapping (Approval Number: GS(2019)1822).

Beijing, the national capital of China, plays a pivotal role in national politics, economy, culture, and international exchanges. Beijing’s central urban area comprises Dongcheng District, Xicheng District, Chaoyang District, Haidian District, Fengtai District, and Shijingshan District (Figure 1). While serving as the functional core of Beijing, this region constitutes merely 8.4% of the total land area yet concentrates key urban resources, including public service facilities, commercial establishments, and cultural institutions. The Seventh National Population Census (2020) showed that the permanent population of Beijing’s central urban area reached approximately 10.93 million, accounting for 50.2% of Beijing. Notably, Report on the Development of Aging Programs in Beijing (2021) highlighted that Beijing has entered a stage of moderate aging, with the older adults in these central urban area growing significantly. In 2020, the adults aged 60 and above in Beijing’s central urban area accounted for 55.34% of the total older adults in Beijing. Specifically, the aging rates of Dongcheng District, Xicheng District, Chaoyang District, Haidian District, Fengtai District, and Shijingshan District were 26.46, 25.97, 20.53, 18.46, 23.71, and 24.26%, respectively. Older adult care facilities within the study area are primarily categorized into three complementary types: older adult care institutions (high-end integrated medical-nursing facilities), older adult care centers (community-based daytime care services), and community older adult care service stations (flexible home-based care for nearby older adults). Against the backdrop of this severe aging challenge, research on the supply and optimal allocation of older adult care resources in Beijing’s central urban area is imperative. Such research also offers valuable insights for the development of the national older adult care service system.

### 2.2 Data resources

This study focused on the Beijing’s central urban area, adopting a 100 m grid as the minimum spatial analysis unit. The core research data consisted of two categories: older adults data and older adult care facility data.

The point-based older adults data utilized in this study was derived from the 2018 “Targeted Assistance” Special Census of the Older Adults in Beijing, a joint initiative by four municipal-level authorities: the Beijing Municipal Civil Affairs Bureau, Beijing Women’s Federation, Beijing Disabled Persons’ Federation, and Beijing Municipal Committee for Older Adults. Census results indicated that the total older adults in central Beijing reached 2.9446 million in 2018. To verify data reliability, cross-validation was conducted against the older adults data (2.3788 million) of Beijing’s central urban area from the 7th National Population Census (2020). The consistency between the two datasets in terms of population scale and spatial distribution patterns confirmed the high reliability of the 2018 older adults data employed in this study. In contrast to the sub-district-level aggregated older adults statistics from previous censuses, the point-based data in this study offered a distinct scale advantage: it enabled the accurate retrieval of the latitude and longitude coordinates of individual older adults, facilitating the refined delineation of their spatial distribution.

Older adults care facility data encompassed three core types of older adult care service providers: older adult care institutions, older adult care centers, and community service stations for older adults. Sourced from the Beijing older adult care facilities full-coverage census in 2020—jointly conducted by the Beijing Municipal Civil Affairs Bureau and the Beijing Jingmin Institute of Social Welfare. This dataset systematically compiled key attribute information of various older adult care facilities, including core indicators such as facility type, specific geographical location and operational status. It comprehensively depicts the spatial layout and supply structure of older adult care facilities in the study area, thereby providing comprehensive facility-side data support for the spatial equity assessment of older adult care facility accessibility.

### 2.3 Methodology

#### 2.3.1 Tyson polygon—determination of service scope

**Figure 2:**
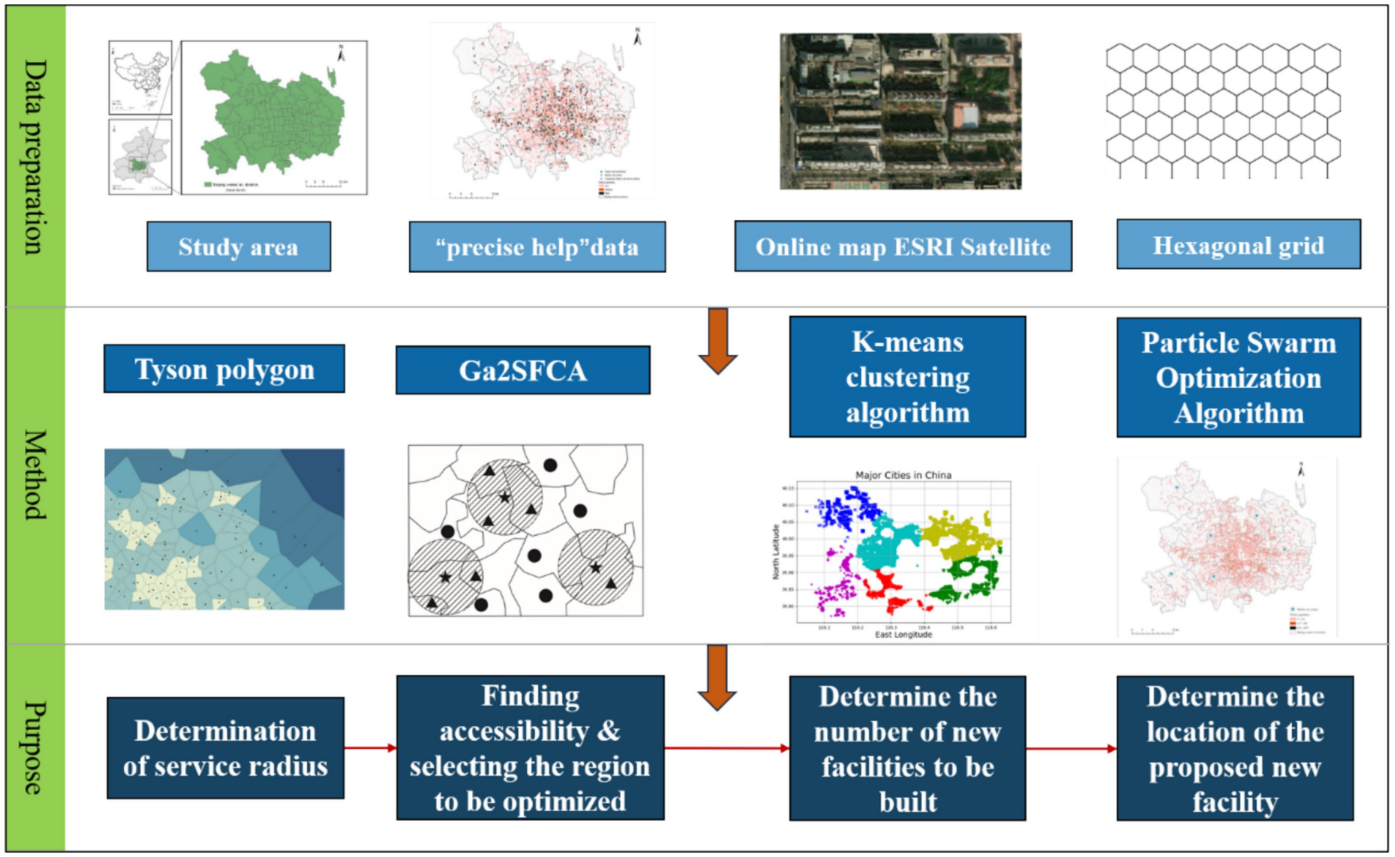
Framework diagram.

The service radii for the three types of facilities (older adult care institution, older adult care center, community older adult care service stations) were determined based on a dual-validation approach combining the geometric characteristics of Tyson Polygon and Beijing’s older adult care service planning standards. Tyson polygon spatial analysis technique (23) was applied to construct the service radius centered on the older adult care facilities. The data grid of the central urban area of Beijing was gridded into a 100 m hexagonal grid in QGIS to ensure uniformity and reduce boundary effects. Then, the corresponding data were connected in ArcGIS to create Tyson polygons for the entire area of Beijing (Figure 2). The Tyson polygons of the central urban area were cropped out, and the radius of the outer circle was calculated. The outer circle’s average radius for community older adult care service stations was calculated as 998 m, which is highly consistent with the “15-minute community life circle” policy (24). Therefore, the service radius for the community older adult care service stations was set to 1,000 m, a standard widely adopted in related empirical studies (25). The service radius of older adults care center was set to 2,500 meters, partly due to the average radius of its Tyson polygon circumcircle being 2,516 meters, and partly considering its coverage as a street/township service center. The service radius of older adult care institutions was set at 3300 meters. In addition to geometric features, it is also a reasonable willingness threshold for older adults to obtain specialized institutional care by taking public transportation for about 30–45 min.

#### 2.3.2 Ga2SFCA

Ga2SFCA calculates the accessibility of public service facilities in two steps based on supply places and demand places, respectively (19). Its specific calculation steps are as follows: the Step 1: take the center of gravity of each supply place *j*, selected or assumed a spatial distance *d_0_*, the formation of its spatial domain, calculate the number of demanders falling within the domain of each demand point *k*, drawing on the Gaussian equation to give weight to and will be added up to get the number of potential demanders of the supply place *j*, and then use the area of the supply place *j* divided by the total number of its potential demanders to calculate the ratio of supply to demand *R_j_* (Equation 1):


$$R_{j}=\frac{S_{j}}{\sum_{k\in \{d_{\mathrm{kj}}\leq d_{0}\}}G(d_{\mathrm{kj}},d_{0})P_{k}}$$ (1)

$$G(d_{\mathrm{kj}},d_{0})=\{\begin{array}{c} \frac{e^{-\frac{1}{2}\times {\left(\frac{d_{\mathrm{kj}}}{d_{0}}\right)}^{2}-e^{-\frac{1}{2}}}}{1-e^{-\frac{1}{2}}},d_{\mathrm{kj}}\leq d_{0}\\ \;\;0\;,d_{\mathrm{kj}}>d_{0} \end{array}\}$$ (2)

where: *d_kj_* is the distance between demand point *k* and supply place *j*; the key Gaussian parameters *d_0_* are set as follows: 1000 m for community older adult care service stations, 2,500 m for older adult care centers, and 3,300 m for older adult care institutions based on the analysis in section 2.3.1; *P_k_* is the number of demanders (i.e.*d_kj_ ≤ d_0_*) in the search area; *S_j_* is the total supply at point *j*; and *G*(*d_kj_, d_0_*) is the distance decay function of the influence of the point source element on the spatial element, i.e., the Gaussian Equation 2.

Step 2: For each demand place *i*, given the spatial distance *d_0_*, its spatial domain is formed, the supply–demand ratio *R_l_* of the supply place *l* that falls within this domain is given a weight using Gaussian equation, and then the weighted ratios are summed to obtain the spatial accessibility of demand place *i*, $A_{i}^{F}$ (Equation 3).


$$A_{i}^{F}=\sum_{l\in \{d_{\mathrm{il}}\leq d_{0}\}}G(d_{\mathrm{kj}},d_{0})R_{l}$$ (3)

where: *R_l_* is the supply–demand ratio of supply point *l* in the search area of demand place (*i*) (i.e.*d_il_ ≤ d_0_*); *dil* is the distance between demand point *i* and the center of gravity *l* of supply place. The larger $A_{i}^{F}$ is, the better the accessibility is. The spatial accessibility calculated by Gaussian two-step moving search method can be interpreted as the number of public service facilities per capita in the study unit.

By calculating the accessibility of older adult care service facilities, grid points with low accessibility levels and those with a non-zero older adults are identified as areas in need of optimization, and new site locations are planned within these areas.

#### 2.3.3 K-means clustering algorithm

K-means, proposed by Macqueen in 1967, belongs to the unsupervised clustering algorithm category. Its core idea is to randomly initialize multiple cluster centers, then divide the data into several clusters based on these centers, and iteratively update the cluster centers to maximize the differences between clusters and minimize the differences within clusters (26–28). When evaluating the clustering effect, the K-means algorithm uses the principle of minimizing performance indicators, with the sum of squared errors (SSE) from intra-cluster points to the cluster centers being the commonly used indicator (29). The elbow rule is a method to evaluate the K-means clustering results using SSE as a cost function. It assesses how the clustering results change with the number of clusters (30). The formula for SSE is as follows (Equation 4):


$$\mathrm{SSE}=\sum_{i=1}^{k}\sum_{p\in C_{i}}{|p-m_{i}|}^{2}$$ (4)

where, *k* is the number of clusters; *C_i_* is the ith cluster in the clustering result; *p* is the sample point in cluster *C_i_*; *mi* is the cluster center of cluster *C_i_*. The elbow method determines the optimal number of clusters according to the SSE degradation based on the improvement effect of SSE, which solves the problem that K-means needs to preset the *k* value. In terms of choosing the number of class clusters, the elbow method will draw the SSE obtained by clustering with different *k* values into a relationship graph, the curve of SSE change with k value is similar to the shape of an elbow, and the position corresponding to the inflection point of the elbow is the optimal number of clusters. The value corresponding to the position with the fastest decrease in SSE during the rise in k-value is the elbow (31).

**Figure 3:**
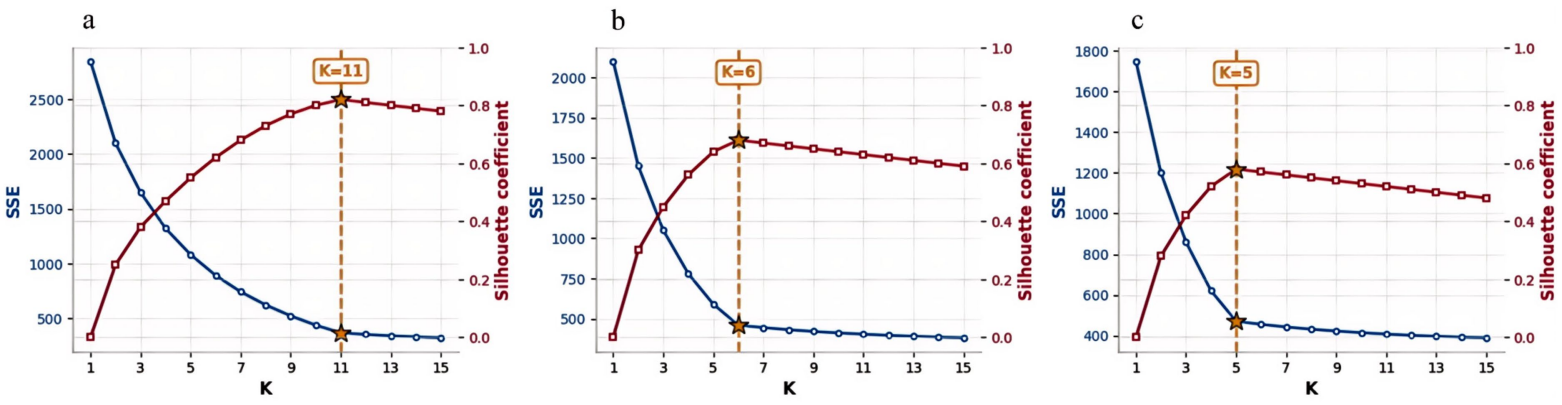
Elbow method for *K*-Means clustering of older adult care facilities (**a**: older adult care institutions, **b**: older adult care centers, **c**: community service stations for older adults).

In ArcGIS, through attribute threshold extraction, extract the raster points with a low accessibility grade and the raster points where the number of older adults is greater than 0 as the area to be optimized. Use QGIS to generate the center points of the grids in the study area and obtain the XY coordinates of each point as the basic data to be processed by the K-means clustering algorithm. The K-means clustering algorithm is programmed in PyCharm for implementation. Input the grid data Excel table containing XY coordinates, obtain the contour coefficients for each number of clusters by running the program, use the elbow rule to determine the number of clusters. The number of selected sites includes 11 community older adult care service stations, 6 older adult care centers, and 5 older adult care institutions (Figure 3). Obtain their clustering classification data tables for each type of older adult care facility, which will be used as the basic data for importing into the PSO algorithm.

#### 2.3.4 Particle swarm optimization algorithm

The Particle Swarm Optimization (PSO) algorithm, proposed by Eberhart et al. in 1995 (32), is an optimization algorithm inspired by the feeding behavior of bird flocks, and the flight process of bird flocks can be regarded as a process of solving multi-objective optimization problems. (33). The PSO algorithm finds an optimal solution to a problem by simulating the social behavior of bird flocks, treating each possible solution as a particle in the search space and updating the location of particles based on the individual optimal solution (Pbest) and the global optimal solution (Gbest) to update the position of the particle to find the optimal solution. In the older adult care facilities location problem, the PSO algorithm first initializes the particle swarm, defines the objective function as the sum of the distances from the grid points to the particle swarm in the region to be optimized, and considers different types of older adult care facilities as different particle swarms. The particle positions are updated through continuous iteration, and Pbest and Gbest are calculated and updated until Gbest meets the termination condition or reaches the maximum number of iterations (34). At this time, the positions corresponding to the particle swarms are the optimal siting positions predicted by the PSO algorithm, which theoretically represent the optimal layout of the older adult care facilities. The details are as follows:

Parameter settings: the number of new facilities n is determined by the K-means clustering results (11 community older adult care service stations, 6 older adult care centers, and 5 older adult care institutions). The swarm size $N_{p}$ (i.e., the number of particles, representing the number of candidate solutions evolved in each iteration) is set in the PSOt package; we report it here to ensure reproducibility. The maximum number of iterations (max_iter) is set to 100; the inertia weight w is set to 0.8; and both learning factors ( $c_{1}$ and $c_{2}$ ) are set to 2. After each update, particle positions are restricted to the predefined optimization boundary.

Initialization of particle positions and velocities: the initial positions and velocities of the particles are randomly generated within a range of values to ensure that they cover the entire optimization region. The rules for updating particle velocity and position are as follows (Equations 5, 6):


$$v_{i}=w\cdot v_{i}+c1\cdot r1\cdot ({\text{pbest}}_{i}-x_{i})+c2\cdot r2\cdot (\text{gbest}-x_{i})$$ (5)

$$x_{i}=x_{i}+v_{i}$$ (6)

*where, v_i_*: current velocity of particle; *w*: inertia weight; *pbest_i_*: best position of particle *i*; *gbest*: global best position; *r1, r2*: random numbers between [0,1]; *x_i_*: current position of particle *i.* After each update, the position of the particle is restricted to the defined boundary.

Design of the fitness function: the fitness function is a criterion for estimating the merit of the particle position. In this study, the fitness function is defined as the sum of the distances between the grid points in the region to be optimized and all the particles in the particle swarm. By minimizing the sum of the distances from all particles to all grid points, the algorithm finds the optimal location of the particle swarm so that it can maximize reachability. Under the fixed facility-number setting, minimizing the summed distances over the low-accessibility demand grids is used as a parsimonious proxy for improving local proximity-based service coverage in the target areas. Because particle coordinates are restricted to the predefined optimization domain, the search process incorporates spatial feasibility constraints by construction; remaining real-world constraints (e.g., land use and implementability) are addressed through the subsequent remote-sensing-based screening. Distance calculation (Equation 7): for each grid point (*lat_j_, lon_j_*) and each particle (*lat_i_, lon_i_*). Fitness value (Equation 8): the fitness value is the sum of distances from all particles to all grid points:


$$d_{\mathrm{ij}}=\sqrt{{\left({\mathrm{lat}}_{i}-{\mathrm{lat}}_{j}\right)}^{2}+{\left({\mathrm{lon}}_{i}-{\mathrm{lon}}_{j}\right)}^{2}}$$ (7)

$${\text{fitness}}_{i}=\sum_{j=1}^{m}d_{\mathrm{ij}}$$ (8)

where, (*lat_j_, lon_j_*) is each grid point, (*lat_i_, lon_i_*) is each particle, *m* is the number of grid points.

On the basis of determining the number of the proposed three types of older adult care facilities, the PSOt package is called in Matlab to realize the PSO algorithm, and the classification result table obtained by the K-means clustering algorithm is substituted into the PSO algorithm code in order to get the location points of each type of older adult care facilities to be built.

## 3 Results

### 3.1 Distribution of older adults and older adult care facilities

**Figure 4:**
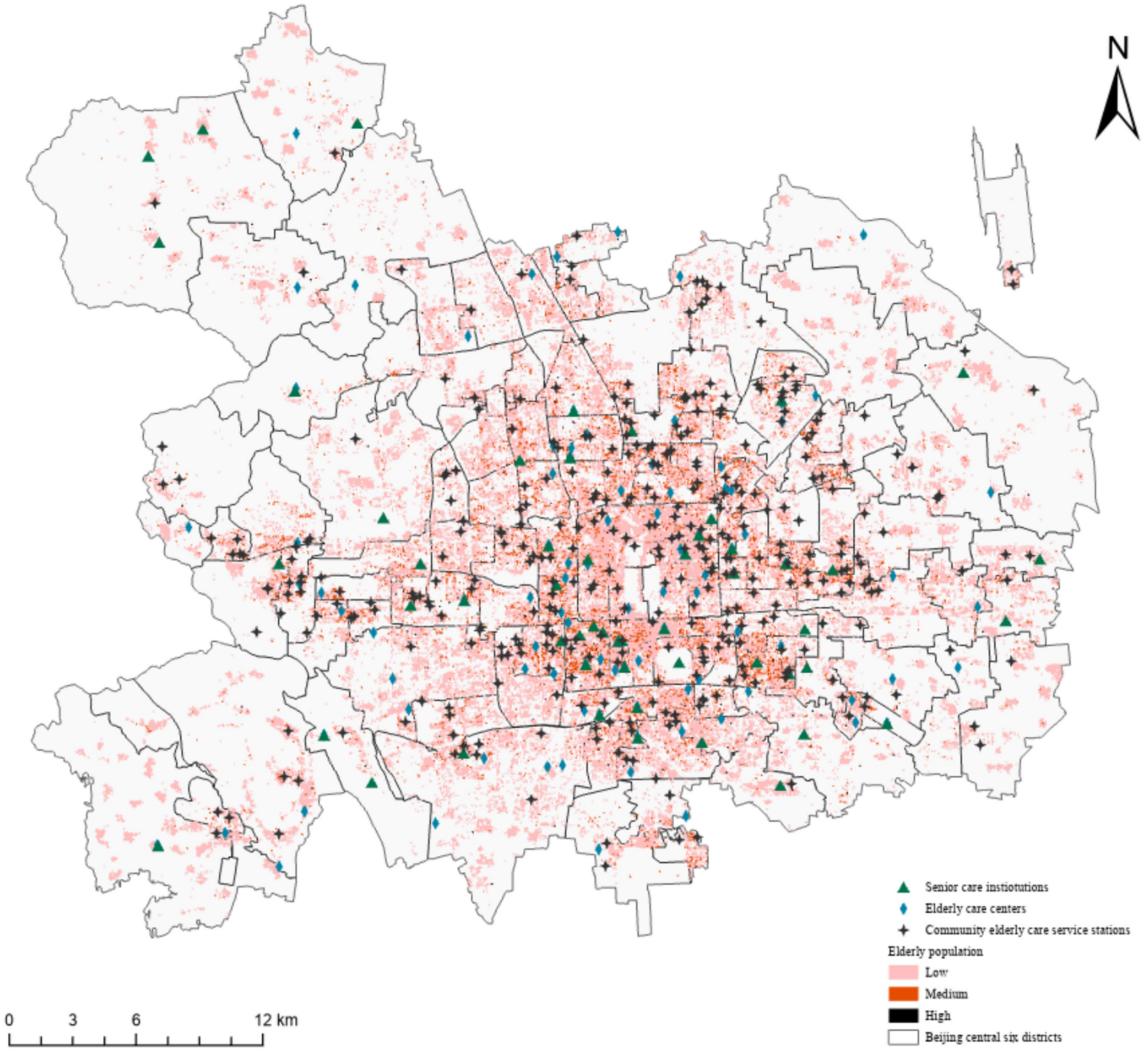
Distribution of older adults and care facilities.

Based on the 100-meter grid to draw the distribution map of older adults and the three types of older adult care facilities in the Beijing central central urban area (Figure 4), it can be seen that the coverage of older adults in these districts is extremely wide. The high-density areas of older adults are obviously concentrated in the central region, while the density around the Beijing central central urban area is relatively low. There are some individual grids in each district with more than 655 older adults, indicating a large demand for older adult care facilities. At present, the number of the three types of older adult care facilities in the Beijing central central urban area accounts for 43% of the total number in the city, including 367 community older adult care service stations, 109 older adult care centers, and 53 older adult care institutions, which account for 42, 60, and 31% of the total number of older adult care facilities in the city, respectively. As can be seen from the distribution of older adult care institutions, older adult care centers, and community older adult care service stations, community older adult care service stations, as the foundation of home-based older adult care services, are an important carrier and the main way for the government to provide basic older adult care services for older adults in the community. They serve as the “service housekeepers” at the doorsteps of older adults and are therefore much more numerous than the other two types of older adult care facilities. The distribution pattern of these facilities is more consistent with that of older adults. In places like Xicheng District’s Guang’anmenwai Street, Chaoyang District’s Wangjing Street and Asian Games Village Street, Shijingshan District’s Bajiao Street, and others, the number of community older adult care service stations constructed is more than five, indicating a higher overall construction density. According to the Beijing City Pension Facilities Construction Plan (35), each street in Beijing City should have a care center for older adults. However, in the periphery of the Beijing central central urban area, there are still some streets that have not yet built older adult care centers. The distribution density of older adults in these areas is low, with lower demand, and part of the functions are replaced by community older adult care service stations. These areas have fewer older adult care facilities than the other two types, and a small number of streets are still not covered.

Looking at the three types of older adult care facilities comprehensively, Wangzuo Town in Fengtai District, Guangning Street in Shijingshan, Qinglongqiao Street in Haidian District, Xibeiwang Town in Haidian District, Shangdi Street in Haidian District, and Sunhe Township in Chaoyang District still only have a single older adult care facility available for older adults at the moment. Additionally, Cuigezhuang Street in Chaoyang District and Xisanqi Street in Haidian District have not yet constructed any older adult care facilities.

### 3.2 Accessibility analysis

**Figure 5:**
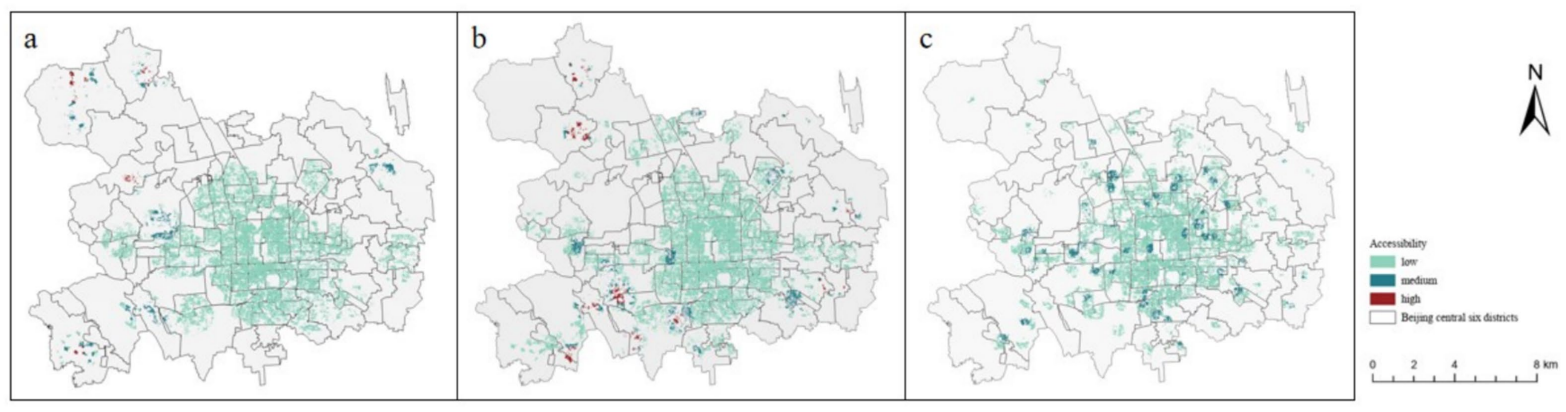
Accessibility of older adult care facilities (**a**: older adult care institutions, **b**: older adult care centers, **c**: community service stations for older adults).

The higher the value of accessibility, the better the accessibility. By calculating the accessibility of the three types of older adult care facilities, we find that the values are in descending order: community older adult care service stations, older adult care institutions, and older adult care centers, with older adult care centers having the lowest accessibility. When we calculate the accessibility of these three types and classify them into high, medium, and low categories, we can observe from the distribution map that within the range of accessibility (Figure 5), the accessibility of the three types of older adult care facilities exhibits a high rate of coverage and is concentrated in the Dongcheng District and the Xicheng District, yet there are still many areas that remain uncovered.

In terms of the community older adult care service stations, the range of high-grade accessibility is extremely limited, appearing only in the Chaoyang District’s Olympic Village Street, Asian Games Village Street, and Shijingshan District’s Wulituo Street. Medium-grade accessibility coverage is higher than that of the other two types of older adult care facilities, and its distribution is more uniform. From the perspective of division levels, older adult care centers have the largest coverage of high-grade accessibility among the three types, with high accessibility concentrated in Wangzuo Town of Fengtai District, Wanping City Area, Lugouqiao Street, Xincun Street, Hot Spring Town of Haidian District, and Shangzhuang Town of Haidian District. Medium-level accessibility is also distributed in their surroundings. Older adult care facilities have the largest service scope, leading to high accessibility in some areas where the older adult is relatively small but older adult care facilities are constructed, such as Wangzuo Town of Fengtai District, Sujiatuo Town of Haidian District, Shangzhuang Town of Haidian District, and Xiangshan Street of Haidian District, all of which are located outside the central urban area of Beijing, in the peripheral areas of Beijing’s central central urban area.

### 3.3 Optimizing the location of older adult care facilities

#### 3.3.1 Determination of the number and location of the proposed older adult care facilities

**Figure 6:**
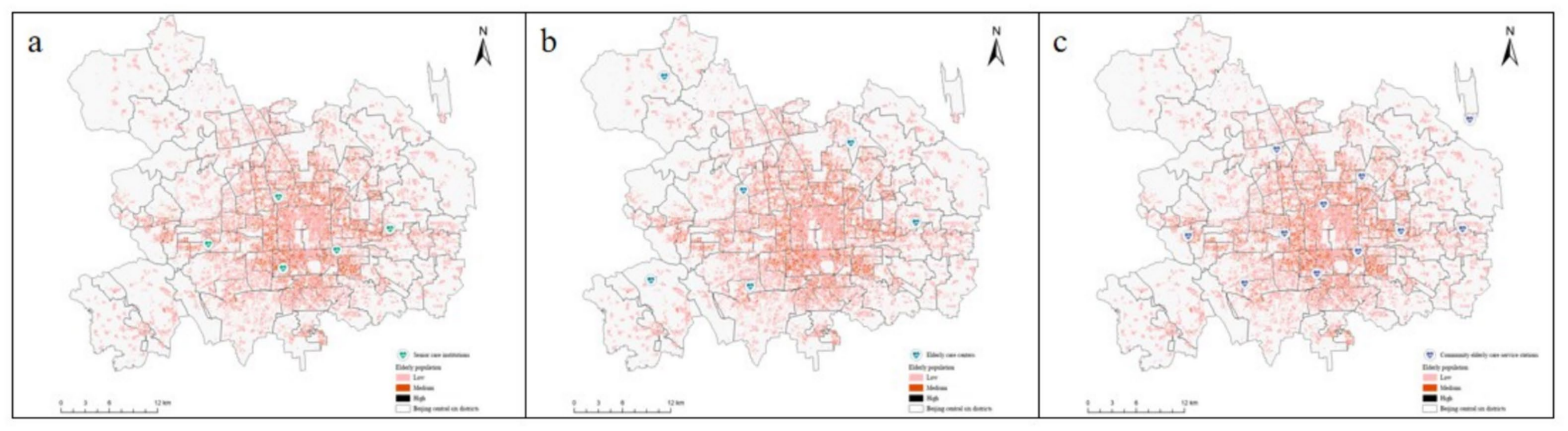
Proposed new older adult care facilities (**a**: older adult care institutions, **b**: older adult care centers, **c**: community service stations for older adults).

After the accessibility evaluation, it can be found that the current accessibility of the older adult care facilities in the central urban area of Beijing is not good. In the face of the continuous growth trend of the older adult, it is urgent to optimize the construction and increase the layout density. According to the results of the accessibility calculation, the grid points with a low accessibility level and the grid points with a older adult greater than 0 are regarded as the areas to be optimized, and new site locations are planned in these areas. With the help of the K-means clustering algorithm and the elbow rule, the number of optimal siting points is determined; then the particle swarm optimization algorithm is used to determine the location of the siting points (Figure 6); and finally, combined with remote sensing images, a feasibility analysis of the theoretical siting locations is conducted.

#### 3.3.2 Feasibility analysis of site location

When determining the location of new older adult care facilities, in addition to maximizing their service effectiveness, it is also necessary to consider the actual situation of urban construction. In order to verify the theoretically optimal siting location, the feasibility is studied from remote sensing images. The feasibility of site selection was analyzed by using the online map ESRI Satellite image in QGIS software and according to the requirements of the Beijing Special Plan for Aging Services (2021–2035; Table 1).

**Table 1:** Statistics on the locations of proposed new older adult care facilities.

Type	No.	Image	Location	Feasibility
Older adult care institution	A1	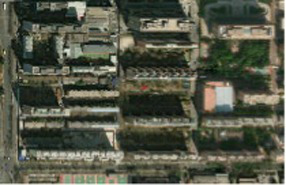	No.40 Fuxing Road, Haidian District	Yes
	A2	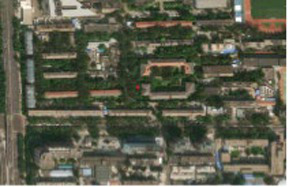	Beijing University of Posts and Telecommunications, Haidian District	No
	A3	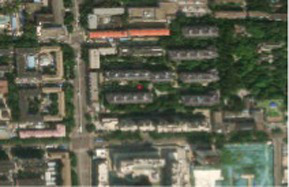	Wanhe Shijia, Xicheng District	Yes
	A4	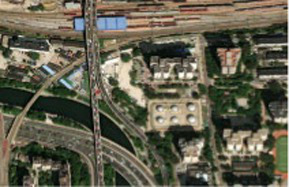	West side of Zhongshili neighborhood, Tonghuihe North Road, Xicheng District	Yes
	A5	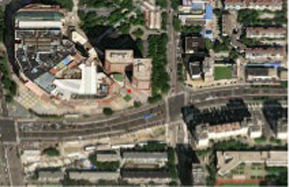	Near Chaoyang Joy City	Yes
Older adult care center	B1	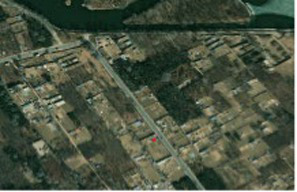	Yujin East Road, Sujiatuo Town, Haidian District	Yes
	B2	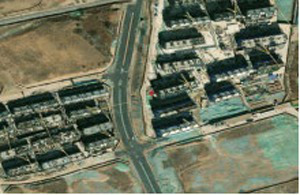	Xinzhuang Village Shantytown Reconstruction Resettlement House, Changxindian Town, Fengtai District	Yes
	B3	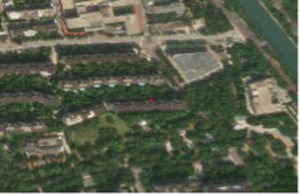	Qingxue Garden, Indigo Factory, Haidian District	Yes
	B4	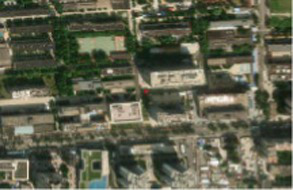	No.20 House, East Street, Fengtai District	Yes
	B5	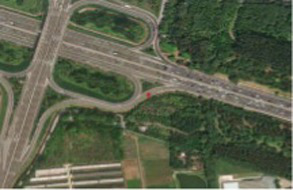	Gujiazhuang Bridge, North Fifth Ring Road, at an important transportation hub	No
	B6	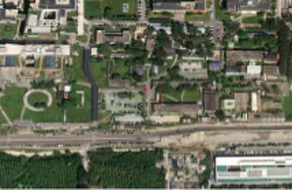	Huitong Times Square, Chaoyang District	No
Community service stations for older adults	C1	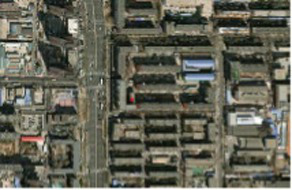	No.5 Courtyard, Gucheng Xiaojie, Shijingshan District, Special Steel Community	Yes
	C2	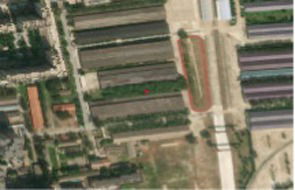	Fengtai District, military land	No
	C3	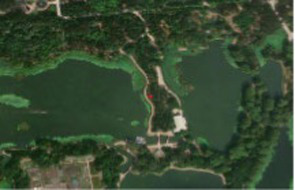	Yuanmingyuan Park, Haidian District	No
	C4	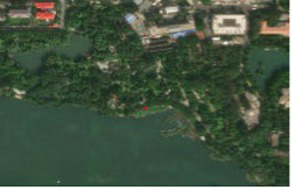	Yuyuantan Park, Haidian District	No
	C5	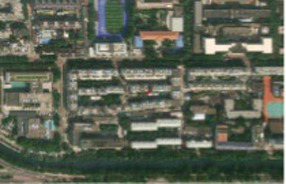	Ande Xinju, Xicheng District	Yes
	C6	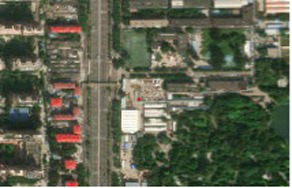	Side of Qing Zhi Yuan, Xicheng District	Yes
	C7	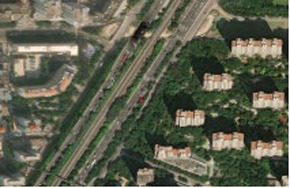	North Side of Sun Gongyuan, Chaoyang District	Yes
	C8	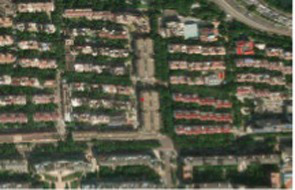	Donghuashibeili Middle Area, Dongcheng District	Yes
	C9	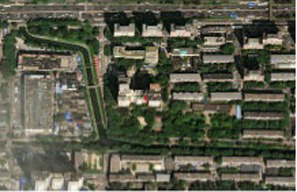	Bishui Xingge, Chaoyang District	Yes
	C10	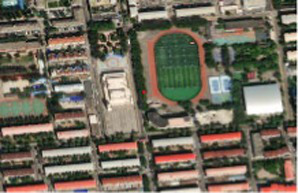	Side of Capital Airport Stadium, Chaoyang District	Yes
	C11	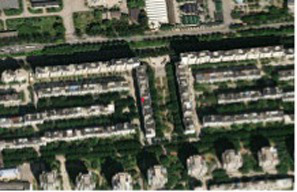	Berliner Philharmonie 4, Chaoyang District	Yes

According to the above empirical study, it can be found that the site location solved by the K-means algorithm and PSO algorithm is relatively reliable, and the site location is basically located in the residential area, and there are potential service recipients within the radius of the site, which can provide services for older adults to a certain extent. However, when determining the site selection, in addition to considering the reliability of the site location and the coverage of service recipients, it is also necessary to comprehensively consider the degree of support of the surrounding community, transportation accessibility, the degree of support of planning policies and other factors, so the above results are only for reference, and the specific construction should be combined with the actual situation.

## 4 Discussion and conclusion

This study shares with previous studies in several ways the concern that population aging is a global social phenomenon, especially in large urban centers, where it is particularly prominent and poses a serious challenge to the socio-economic and aging services systems. Previous studies have extensively explored this topic, and this study builds on this with a more in-depth analysis. Second, both focus on the layout optimization of aging services facilities, aiming to enhance the accessibility and coverage of aging services through scientific methods. This focus on the optimization of facility layout reflects the common pursuit of improving the quality of life of older adults and promoting the balanced development of aging services. Finally, in terms of research methodology, this paper also draws on spatial analysis tools commonly used in previous studies, the application of which provides strong support for an in-depth understanding of the spatial relationship between older adult care facilities and older adults.

We compared our approach and results with representative studies on public service facility accessibility in major Chinese cities (Beijing, Shanghai, Shenzhen and Jiande). Table 2 summarizes the differences in data, radii, algorithms, and validation. The combined use of Ga2SFCA, K-means, and PSO has significant advantages in evaluating accuracy and policy application than existing single-method. The service radius setting of Ga2SFCA in measuring accessibility was similar to that of other scholars. The framework utilized K-means clustering to identify the optimal number of new facilities based on the actual spatial aggregation of the underserved older adults with elbow rule. This ensured that the quantity of proposed facilities was data-driven rather than administrative. Furthermore, considering conventional siting models may fall into local optima, the PSO algorithm performed a global search to identify coordinates that minimize the aggregate distance for the clustered demand. This combination ensures that the proposed sites were mathematically optimal for the specific population distribution of Beijing’s central urban area. The “equity-oriented” of this study moved beyond simple facility coverage. By using Ga2SFCA on a 100 m grid, we identified “blind spots” that aggregate statistics miss. The subsequent optimization did not merely fill gaps but prioritizes high-density, low-accessibility areas, aligning with the “Targeted Assistance” policy goal of the Beijing government. Moreover, this study differentiated between facility types. The integrated framework provided a multi-solution: determining precise locations for community stations and strategic zones for institutions. This layered output offered urban planners a direct, executable “roadmap” for resource allocation. The conclusions significantly improved the efficiency of public health spending compared to uniform allocation strategies. At the same time, the optimized allocation of older adult care facilities in Beijing’s central urban area proposed in this study will help promote spatial equity in older adult care services, improve older adults’ life quality and social well-being, and contribute to building a harmonious and livable urban environment.

**Table 2:** Comparison of related research on public facility accessibility and optimization.

Study area	Data level	Radii strategy	Optimization algorithm	Validation method
Beijing (This Study)	2018 point-based population and 2020 older adult care facility	Service radius of community older adult care service stations, older adult care center and older adult care institution were 1,000 m 2,500 m 3,300 m.	K-means + PSO	Remote sensing imagery feasibility check
Beijing (17)	2010 street-level population and 2013 older adult care facility	Service radius of small, medium, and large older adult care facilities were 0.5 h, 1 h, and 2 h.	N	Comparative analysis of different facilities
Shanghai (15)	2015 street-level population and 2013 older adult care facility	Service radius of small, medium, and large older adult care facilities were 30, 60, 90 min.	N	Spatial autocorrelation analysis
Jiande (36)	2020 street-level population and 2020 facility poi data	“5–10–15 min” life circle, walking distance thresholds were set at 300–500–800 meters.	Coverage gap identification	Questionnaire & Field visit
Shenzhen (7)	2017 mobile phone population and 2018 park facility	Hierarchical radii for park types	K-means Clustering	Policy implication analysis
Shenzhen (37)	2020 street-level population and 2021 medical facility	Radius of search area for facilities	Maximum Equity Model	Statistical distribution analysis

Their is an observed paradox of high older adults density coupled with low facility accessibility in Beijing’s central urban area. This disparity is not merely a result of planning insufficiency but is fundamentally driven by structural constraints, including land scarcity, prohibitive development costs, and strict preservation policies within the capital’s core. Consequently, the dense aging population in these areas remains underserved. We argue that the current strategy must shift from large-scale expansion to precision embedding to support the “9,073” pattern. Our optimization results (Figure 6) precisely identify the specific “blind spots” where these facilities should be strategically inserted. While the siting results are policy-aligned, their practical integration pathways remain underdeveloped. This study proposed a implementation framework focusing on space, funding, and management to bridge the gap between theoretical optimization and real-world application. First, regarding spatial implementation, an embedded land use strategy should be prioritized to overcome land scarcity. The implementation of the proposed 16 new facilities should rely on urban regeneration and stock utilization. Planners should prioritize retrofitting underutilized spaces near the siting results, such as vacated industrial plants or inefficiently used community administrative offices. This embedded approach allows community older adult care service stations to be integrated into the existing urban fabric, minimizing displacement and construction costs. Second, regarding operational feasibility, a diversified investment mechanism leveraging Public-Private Partnerships (PPP) is essential. The optimization results identify areas with high demand but low supply, which may initially seem less profitable for private investors. To address this, the government should provide targeted policy incentives—such as rent subsidies, tax reductions, or construction grants—specifically for facilities established in the identified optimization zones. A cooperative model where the government provides the venue and social enterprises provide professional services can ensure the long-term sustainable operation of these newly sited facilities. Third, regarding governance, a dynamic smart monitoring platform should be established based on the grid GIS framework. The spatial distribution of older adults is not static. The grid-based infrastructure built should be upgraded into a dynamic management system. By integrating real-time data from the civil affairs and health departments, urban planners can continuously monitor fluctuations in the supply–demand ratio within each grid. This allows for the flexible adjustment of service functions within the proposed facilities.

However, this study also has some limitations. First, regarding data timeliness, this study utilized point-based older adults data from 2018. Although this dataset offers precise spatial resolution crucial for micro-level accessibility assessment and siting optimization, it may not fully capture the demographic changes after 2018. Considering older adults care facility data from 2020, the mismatch between population and facility data may lead to deviation. Especially after the outbreak of COVID-19, older adults care needs may change, which has not been discussed. Second, the accessibility evaluation in this study only considered the spatial distance, did not take into account the influence of factors such as the transportation accessibility, travel mode and land-use constraints, while did not conduct sensitivity test. Finally, the results of the optimal siting need to be further verified and adjusted in the context of the actual situation to ensure their feasibility and effectiveness. Future research could further enhance accuracy by integrating multi-source dynamic data, such as real-time mobile signaling data or the latest community grid management data, to conduct sensitivity analyses and dynamically monitor changes in accessibility. At the same time, we can further explore different service radius effects and improve the accessibility evaluation method, incorporating more factors into the evaluation system, such as multi-modal transportation networks and real-time traffic data, so as to improve the accuracy and practicality of the evaluation results. In addition, strengthening empirical research is also one of the important directions for future research. Through the collection and analysis of field research and social survey data, we can verify and adjust the results of optimal site selection to ensure that the research results can be truly implemented and achieve practical results, and provide a more scientific basis for decision-making in urban aging management.

## Data Availability

Publicly available datasets were analyzed in this study. This data can be found at: https://microdata.stats.gov.cn/. The point-based older adults data utilized in this study was derived from the 2018 “Targeted Assistance” Special Census of the Older Adults in Beijing. Older adults care facility data was sourced from the Beijing older adult care facilities full-coverage census in 2020—jointly conducted by the Beijing Municipal Civil Affairs Bureau and the Beijing Jingmin Institute of Social Welfare.
